# Fabrication and Characterization of Carbonate Apatite-Bovine Gelatin Scaffolds for Endodontic Regeneration: A Lyophilization-Based Approach

**DOI:** 10.1055/s-0045-1809306

**Published:** 2025-05-29

**Authors:** Ratih Widyasari, Arief Cahyanto, Sunardhi Widyaputra, Arif Rachman

**Affiliations:** 1 Doctoral Programme, Faculty of Dentistry, Padjadjaran University, Sumedang, Indonesia; 2 Department of Conservative Dentistry, Faculty of Dentistry, Universitas Jenderal Achmad Yani, Cimahi, Indonesia; 3 Department of Clinical Sciences, College of Dentistry, Ajman University, Ajman, United Arab Emirates; 4 Centre of Medical and Bio-allied Health Science Research, Ajman University, Ajman, United Arab Emirates; 5 Department of Oral Biology, Faculty of Dentistry, Padjadjaran University, Sumedang, Indonesia; 6 Department of Oral Biology, Faculty of Dentistry, Maranatha Christian University, Bandung, Indonesia; 7 Department of Cell Biology and Biomolecular, Faculty of Medicine, Indonesian Defense University, Bogor, Indonesia

**Keywords:** bioactivity, bovine gelatin, carbonate apatite, crystallinity, endodontic regeneration, lyophilization, scaffold

## Abstract

**Objectives:**

The challenge of endodontic regeneration requires scaffold that can facilitate dentin and pulp regeneration by providing structural support and promoting initial cell adherence to regenerate new tissue. This study explores characterization of a novel carbonate apatite-bovine gelatin (CA-BG) scaffold for endodontic regeneration that was fabricated using a lyophilization technique. CA, recognized for its biocompatibility and osteoconductive capabilities as a scaffold, was expected to provide structural support in complex biological environments such as pulp tissues. BG, a natural polymer with cell attachment substrates, was incorporated into the scaffold to enhance bioactivity, promoting cell attachment, proliferation, and differentiation.

**Materials and Methods:**

Scaffolds were fabricated with varying liquid-to-powder (L/P) ratios (0.5, 0.8, and 1) using freeze drying, and then their chemical and structural properties were evaluated using Fourier-transform infrared spectroscopy (FTIR), X-ray diffraction (XRD), and scanning electron microscopy (SEM).

**Results and Discussion:**

FTIR analysis confirmed the presence of carbonate and phosphate groups, with slight peak shifts indicating CA-BG interaction. XRD analysis showed crystallinity differences, which were affected by the liquid ratio in each group. SEM results demonstrated that the L/P 1 scaffold exhibited surface roughness, which is expected to represent BG incorporation to CA. The L/P 1 scaffold was identified as the optimal candidate, balancing bioactivity and structural properties, to be able to promote dentin and pulp tissue regeneration.

**Conclusion:**

The findings contribute significantly to developing biocompatible, bioactive scaffolds for endodontic regeneration and broader tissue engineering applications, offering insights to achieve a balance between a scaffold structure and its biological functionality.

## Introduction

Endodontic regeneration presents a unique challenge in tissue engineering, because of its requirement of scaffold materials that can support both soft and hard tissue regeneration. 1 Various scaffold materials have been explored in endodontics, each with distinct advantages and limitations. Collagen-based scaffolds, for instance, mimic the extracellular matrix and enhance cellular attachment, but their limited mechanical strength and rapid degradation often fail to provide sufficient support for complete tissue regeneration. 2 Synthetic polymers, such as polylactic acid (PLA) and polyglycolic acid (PGA), offer tunable degradation rates and mechanical properties but lack the bioactivity necessary for complex tissue environments like dental pulp. 3 Bioceramics such as hydroxyapatite (HA) and β-tricalcium phosphate (β-TCP) exhibit excellent osteoconductivity and mechanical stability, yet they require integration with bioactive components to promote cellular attachment and proliferation in soft tissue regeneration. 4 Despite progress, gaps remain in achieving consistent and predictable outcomes, as highlighted by Meschi et al, who underscored the lack of sufficient evidence supporting regeneration/revitalization for apical periodontitis in immature permanent teeth. 5

Using scaffolds in regenerative endodontics has shown potential in promoting vascularization, cellular infiltration, and tissue regeneration. Prior work by Palma et al on lyophilized hydrogel chitosan scaffolds demonstrated biocompatibility and vascularization but revealed challenges such as obstruction of apical tissue ingrowth when the scaffold persisted within the canal. 6 Similarly, regenerative procedures relying on antibiotic pastes, reported by 41.7% of endodontists and pediatric dentists in a multinational survey, highlight the limited consensus on optimal materials and protocols in practice. These findings underscore the need for alternative scaffold designs to facilitate cell infiltration and nutrient diffusion while maintaining structural integrity. 7 Another study of endodontic regeneration emphasized how crucial it is to use the proper scaffolds in dental pulp tissue engineering. The study failed to display the odontoblasts and newly formed dentin along the root canal's dentinal wall, which may have been caused by the collagen scaffold contracting and preventing the cells from contacting the dentinal wall. 8

Recent research has shown that combining biomaterials, particularly those involving bioceramics like silica calcium phosphate, have improved scaffold bioactivity and mechanical properties. 9 Studies on incorporating biomaterials have demonstrated increased biointegration and enhanced tissue regeneration potential, primarily through controlled degradation, improved ion release (Ca ^2+^ , PO _4_^3−^ ), and enhanced mechanical strength. Bioglass, recognized for its bioactivity, promotes osteoconduction and accelerates bone formation. 10 Another study on integration of natural polymer such as hydrogel into bioactive glass scaffolds provide the extra spatial dimensions needed to replicate a tissue microenvironment and improve hierarchical cell–cell and cell–matrix interactions. It has also been demonstrated that a three-dimensional structure provides the ideal surface for cell adhesion and proliferation. 11 Gelatin-containing bioceramic scaffolds have been extensively researched for bone and dental regeneration, highlighting gelatin's ability to form hydrogels conducive to cell migration and differentiation. 12 Natural polymer-based scaffolds, including those using chitosan, collagen, and alginate, have successfully mimicked the extracellular matrix and enhanced scaffold bioactivity. 13 Such scaffolds are widely used in tissue engineering because they promote cell adhesion and tissue integration, making them suitable for dental and bone regeneration. 14

Carbonate apatite (CA) has emerged as a promising biomaterial due to its biocompatibility, resorbability, and similarity to the mineral component of natural bone, making it particularly useful as a scaffold in bone tissue regeneration. 9 CA mimics the carbonate content of human bone, which enhances its bioresorption rate compared with HA, making it particularly suitable for tissue engineering applications requiring gradual scaffold degradation and replacement with natural tissue. 15 Its ability to release bioactive ions, such as calcium and phosphate, further supports its role in promoting cell adhesion and differentiation, which is critical for tissue regeneration. However, CA alone may lack the bioactivity required for adequate soft tissue regeneration, highlighting the need to combine it with bioactive materials. 16

Bovine gelatin (BG), a natural polymer derived from collagen, is a biologically active, biocompatible, and biodegradable polymer that can be utilized as an efficient scaffold. 17 18 Gelatin is known for its cell-adhesive properties, making it highly effective in promoting cell attachment, proliferation, and differentiation, which are crucial in tissue regeneration. 19 BG is known for its ability to form hydrogels, creating a favorable environment for cell migration and nutrient exchange. However, its mechanical properties alone are insufficient for load-bearing applications or tissues requiring significant structural support, making it ideal for combination with stronger materials like CA. 17

This study introduces a novel CA-BG scaffold developed using lyophilization technique (freeze drying), designed to achieve balance between porosity, bioactivity, and mechanical stability. Unlike chitosan or single-material scaffolds, CA-BG offers a synergistic combination of CA's osteoconductive and structural properties with BG's bioactivity. This approach addresses limitations observed in prior studies, such as poor vascularization and mechanical instability, by creating a scaffold optimized for gradual degradation and cellular interaction. This work adds to the increasing amount of evidence in this developing field by comparing its performance to current scaffolds and offering fresh perspectives on using biomaterials for endodontic regeneration.

The CA-BG scaffolds were prepared with three different liquid-to-powder (L/P) ratios (0.5, 0.8, and 1.0) to examine how these variations affect their microstructure and crystallinity. The CA-BG scaffolds were compared with the original CA to assess its potential as a novel scaffold material for endodontic regeneration. The study will focus on identifying the optimal L/P ratio that balances porosity, crystallinity, and bioactivity, thereby offering insights into the design of scaffolds that can effectively support the regeneration of dental pulp and surrounding tissues. Furthermore, the findings of this research may extend beyond endodontics, contributing to the broader field of tissue engineering, where the development of biocompatible and bioactive scaffolds remains a critical challenge. The need for scaffold optimization to achieve specific clinical goals has been extensively documented, underscoring the importance of balancing mechanical strength with biological functionality. 20

## Materials and Methods

This study represents the preliminary phase of a more extensive investigation into CA-BG scaffolds. CA was produced internally using a previously defined formulation from vaterite. 21 22 BG used ready product BG Solution 2% (Sigma-Aldrich, Darmstadt, Germany). Teflon molds created in compliance with ISO 9917–1 specifications for water-based cements were used to produce scaffolds. The fabricated CA-BG scaffolds were in three L/P ratios: 0.5, 0.8, and 1, with BG as liquid and CA as powder. After the mixture of CA-BG, the scaffolds were prepared using a freeze-drying technique (Biobase BK-FD10P, Jinan, China). To get the required porosity and structural integrity, the scaffolds were vacuum-dried at 0.05 mbar for 24 hours after being frozen at −20°C for 2 hours. The composition used for this study is presented in Table 1 .

**Table 1:** Composition of scaffold specimens

Group	L (mL)	P (g)	Freezing time	Freeze-drying time
L/P 0.5	0.25	0.5	2 h	24 h
L/P 0.8	0.4	0.5	2 h	24 h
L/P 1	0.5	0.5	2 h	24 h

Abbreviation: L/P, liquid-to-powder ratio.

The characterization of the scaffolds was performed using several Fourier-transform infrared spectroscopy (FTIR), X-ray diffraction (XRD), and scanning electron microscopy (SEM). FTIR was employed to identify functional groups, with spectra recorded in the 400 to 4000 cm ^−1^ range using a Shimadzu spectrometer (Shimadzu, Kyoto, Japan). XRD analysis was conducted to assess the crystallinity of the scaffolds, covering a 2θ range from 3 to 50 degrees using a Bruker D8 Advance diffractometer (Bruker, Ettlingen, Germany). Additionally, SEM was performed using a JEOL JSM-6510LA system (JEOL, Tokyo, Japan) to evaluate the microstructure of the scaffolds at a magnification of 30× and 250 × .

The experimental study involved scaffold preparation and characterization through qualitative assessment methods. Scaffold fabrication was confirmed through repeated evaluations across three experimental runs by two operators, enhancing the reliability of the findings. The experiments were performed three times for each condition to achieve the right consistency of the scaffold. For SEM sample preparation, qualitative assessments of scaffold structure and porosity during SEM sample preparation were performed by two independent observers, with consensus reached to minimize bias. For FTIR and XRD analyses, selected representative samples were analyzed without requiring multiple observers or repeated trials, as these techniques provide standardized, instrument-based measurements.

## Results

### FTIR Analysis

**Fig. 1:**
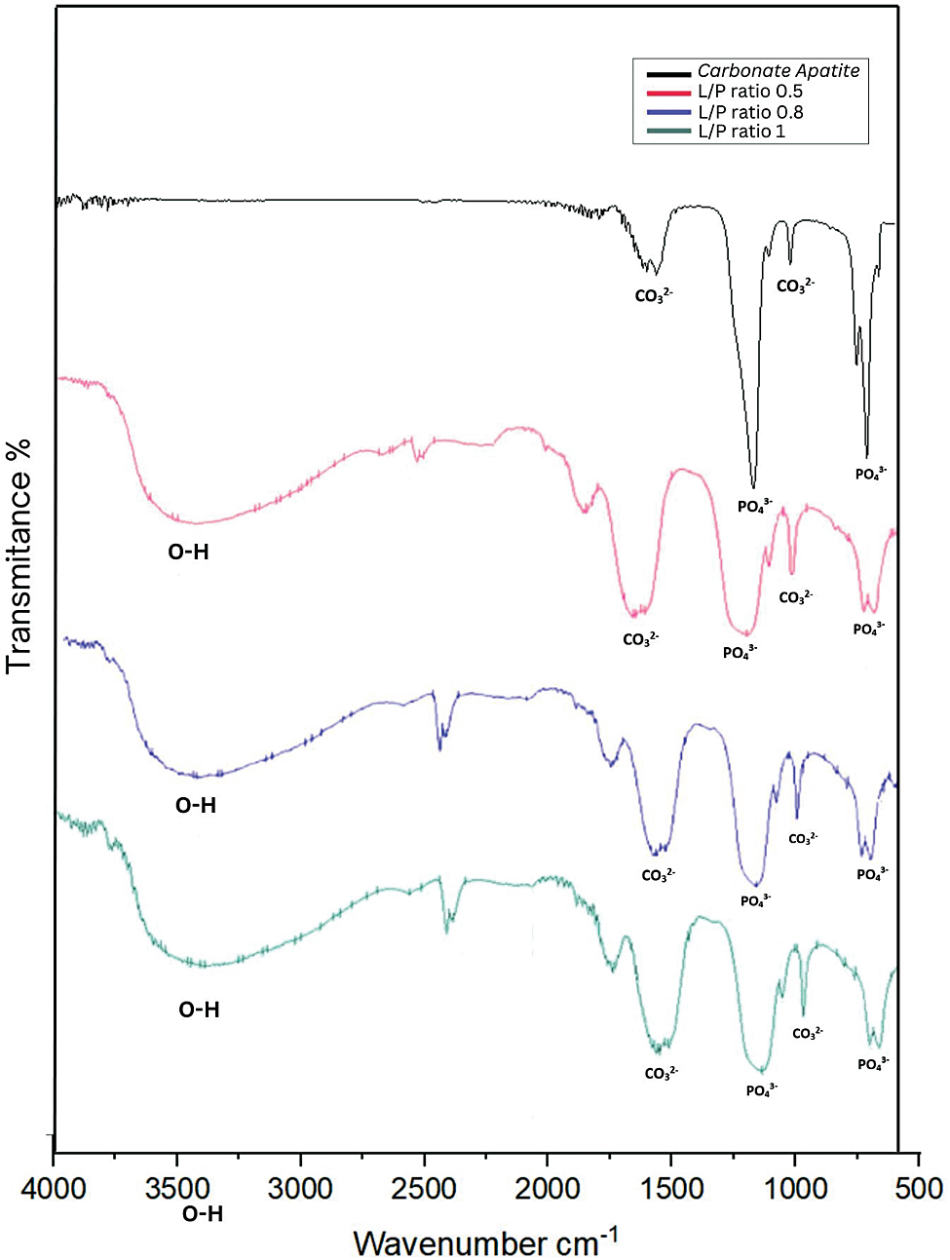
Fourier-transform infrared spectroscopy (FTIR) spectra comparing the carbonate apatite-bovine gelatin (CA-BG) scaffolds and the original CA, with key peaks highlighted.

The FTIR spectra confirmed the presence of characteristic carbonate (1420–1450 cm ^−1^ ) and phosphate (1030–1100 cm ^−1^ ) groups in both the CA-BG scaffolds and the original CA. The addition of BG introduced slight shifts in these peaks, suggesting interactions between CA and BG. These interactions, particularly in the L/P 1 sample, indicated a possible reduction in crystallinity, as further evidenced by the XRD patterns ( Fig. 1 ).

The FTIR spectra of the original CA and the CA-BG scaffold samples are shown in Fig. 1 . The original CA displayed characteristic carbonate (CO _3_^2−^ ) peaks at approximately 1420 to 1450 cm ^−1^ and phosphate (PO _4_^3−^ ) groups around 1030 to 1100 cm ^−1^ . These peaks were present in all CA-BG scaffold samples (L/P 0.5, L/P 0.8, L/P 1), indicating that CA's fundamental chemical structure was retained after adding BG. However, subtle differences were observed between the original CA and the CA-BG samples. In the CA-BG scaffolds, the intensity of the carbonate and phosphate peaks was slightly shorter, particularly in the L/P 1 sample, suggesting a decrease in carbonate and phosphate content due to the higher L/P ratio. The interaction between CA and BG could have an impact on the scaffold's overall structure and composition. In addition, the wavenumbers of a few peaks in the CA-BG samples were marginally different from those in the original CA. This change suggests that there are molecular interactions between the BG and the CA, which supports the BG's incorporation into the scaffold matrix.

### XRD Analysis

**Fig. 2:**
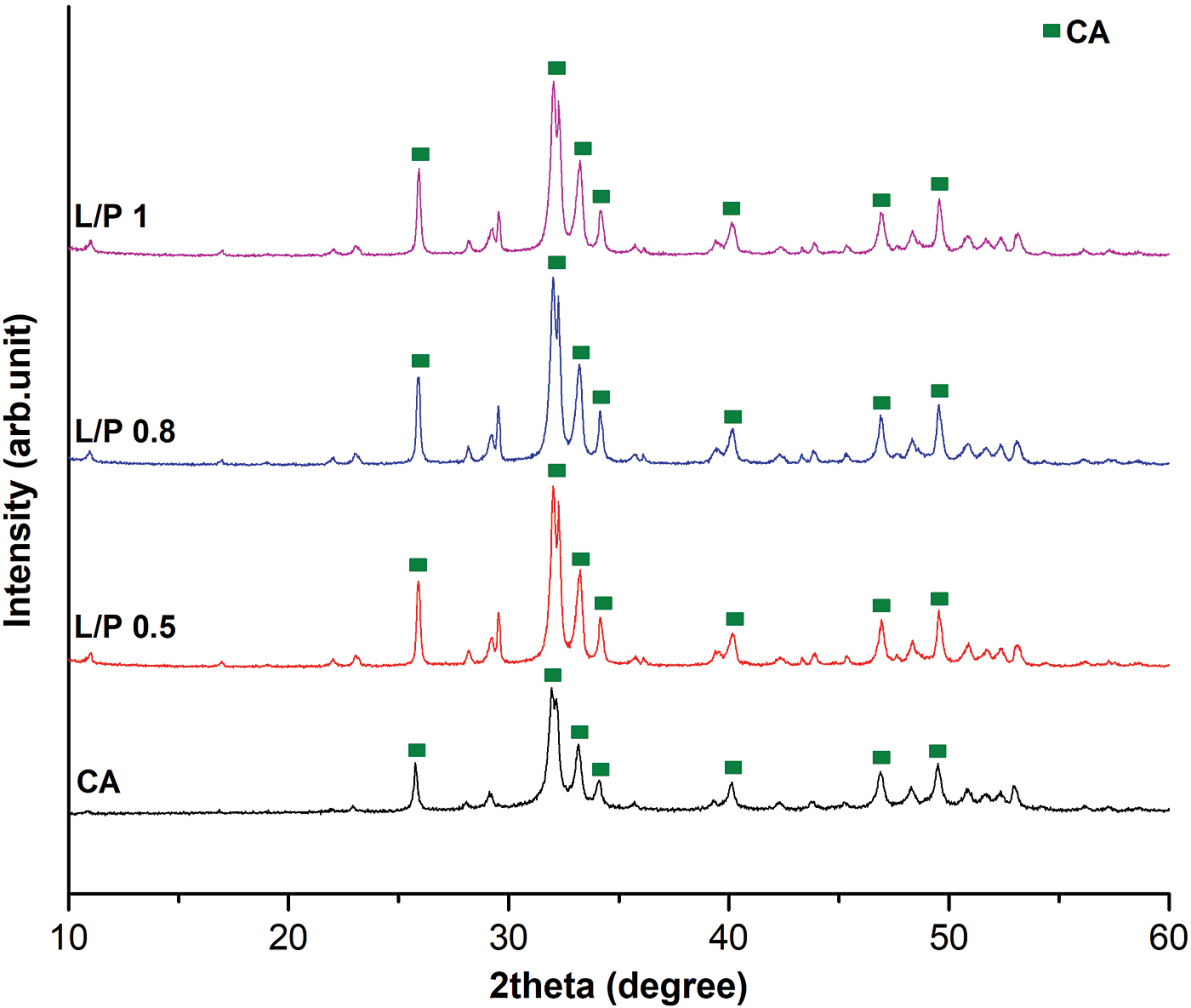
X-ray diffraction (XRD) patterns for carbonate apatite-bovine gelatin (CA-BG) scaffolds and original CA.

XRD analysis showed that the original CA exhibited sharp peaks indicative of high crystallinity, especially in the 2θ range of 26 to 33 degrees. In contrast, the CA-BG scaffolds, particularly L/P 0.8 and L/P 1, displayed broader and less intense peaks, reflecting a decrease in crystallinity. This reduction in crystallinity benefits scaffold resorbability, making CA-BG scaffolds more suitable for tissue engineering applications where gradual scaffold degradation is desired ( Fig. 2 ).

The XRD patterns of the original CA and the CA-BG scaffolds are shown in Fig. 2 . The original CA showed sharp diffraction peaks, particularly in the 2θ range of 26 to 33 degrees, corresponding to the crystalline phases of CA. These peaks were also present in the CA-BG scaffolds, confirming that the CA structure was preserved in all samples. However, the CA-BG scaffolds, especially in the L/P 0.8 and L/P 1 samples, showed broader and less intense diffraction peaks than the original CA. This suggests a decrease in crystallinity with the addition of BG, particularly at higher L/P ratios. The higher peak in the L/P 1 sample indicates a more amorphous structure, which is likely due to the higher liquid content during the scaffold fabrication process.

In contrast, the L/P 0.5 sample showed peak closer to the original CA, indicating that the lower liquid content helped preserve the crystallinity of the material. These differences in crystallinity between the original CA and the CA-BG scaffolds suggest that the incorporation of BG affects the scaffold's microstructure and phase composition.

### SEM Analysis

**Fig. 3:**
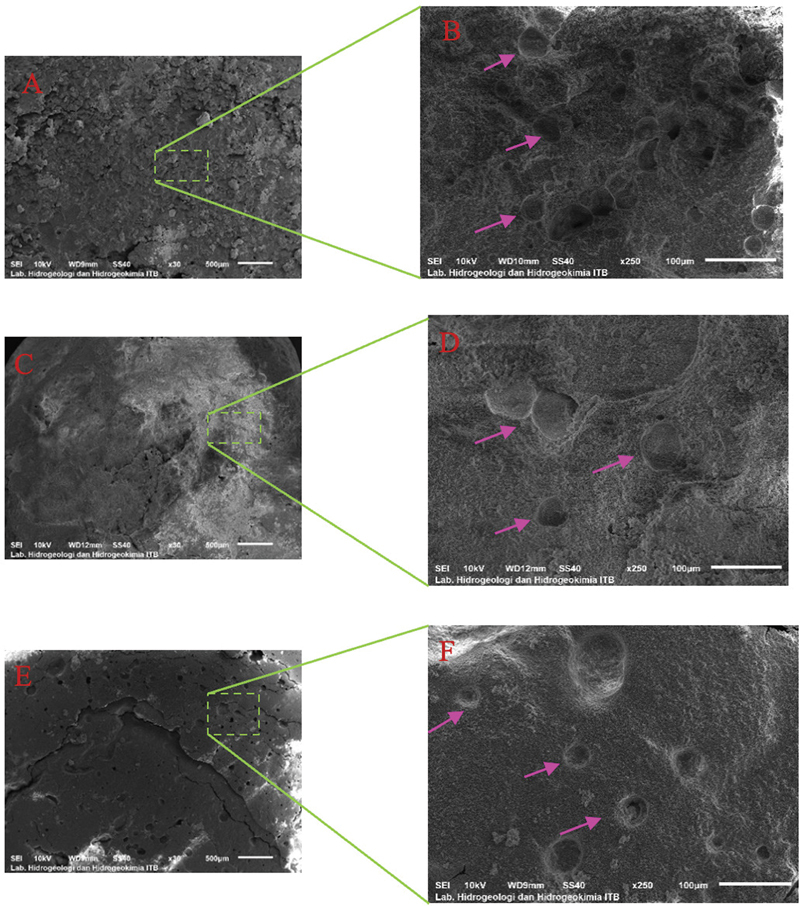
Scanning electron microscopy (SEM) images (30× and 250 × ) of the liquid-to-powder (L/P) 0.5, L/P 0.8, and L/P 1 scaffolds. ( **A** ) L/P 0.5 at 30× magnification. ( **B** ) The L/P 0.5 scaffold has the roughest texture (250 × ). ( **C** ) L/P 0.8 at 30 ×'x magnification. ( **D** ) The L/P 0.8 scaffold shows smoother texture (250 × ). ( **E** ) L/P 1 at 30× magnification. ( **F** ) The L/P 1 scaffold has the smoothest surface (250 × ).

SEM images revealed that the L/P 0.5 scaffold displayed more roughness. In contrast, the L/P 0.8 and L/P 1 scaffolds with higher liquid content demonstrated smoother structures ( Fig. 3 ).

At 30× magnification, the surface structure of the L/P 0.5 scaffold exhibits more roughness. Some craters, shown by green box, are distributed across the scaffold surface. The crater sizes are relatively uniform, with visible roughness on the scaffold surface. The finer structural details at 250× show smaller, more intricately distributed pores. The scaffold in the L/P 0.8 group shows a smoother surface compared with L/P 0.5, with larger pores still present but less uniformly distributed at 30× magnification. The surface texture appears smoother than L/P 0.5. At 250× magnification, the L/P 0.8 scaffold shows fewer craters. The overall texture is less rough, with the scaffold surface appearing to be more compact. The L/P 1 scaffold presents least uniformly distributed craters at 30× magnification. The scaffold surface appears the smoothest of the three groups, suggesting a highly compact structure. At 250× magnification, the scaffold surface shows small numbers of craters with a very smooth texture.

## Discussion

Compared with the original CA, the CA-BG scaffolds significantly reduced crystallinity and surface structure roughness, particularly in every increasing L/P ratio. The L/P 1 scaffold retained crystalline structure but more amorphous, making it the most promising candidate for endodontic regeneration, where scaffold resorption and bioactivity are essential, but the mechanical and structural stability are also playing an important role. The addition of BG likely enhanced the scaffold's bioactivity, promoting cell attachment and proliferation, while the reduced crystallinity allowed better resorbability in tissue engineering applications. 19 23

### FTIR Analysis: Chemical Composition and Interactions

The FTIR spectra confirmed the presence of characteristic carbonate and phosphate peaks in both the original CA and the CA-BG scaffolds. The carbonate bands at 1420 to 1450 cm ^−1^ and phosphate bands at 1030 to 1100 cm ^−1^ were evident in all samples, indicating that the fundamental chemical structure of CA remained intact following the incorporation of BG. On the other hand, slight changes in peak locations within the L/P 1 scaffold imply molecular interactions between BG and CA, most likely caused by hydrogen bonding between the gelatin's carboxyl, amine groups and the surrounding CA matrix. These molecular interactions indicate that BG enhances bioactivity, which is consistent with the literature emphasizing the importance of natural polymers in biomaterial scaffolds. 24 While this study employed a qualitative approach, future investigations could include quantitative analysis of peak intensity ratios to provide objective data on carbonate and phosphate content variations across different formulations.

### XRD Analysis: Crystallinity and Structural Integrity

The XRD patterns highlighted differences in crystallinity between the original CA and the CA-BG scaffolds. The sharp peaks observed in the original CA, particularly in the 2θ range of 26 to 33 degrees, indicated a highly crystalline material. In contrast, the CA-BG scaffolds, especially those with higher L/P ratios, displayed broader and less intense peaks, signifying reduced crystallinity. This reduction in crystallinity is likely due to the incorporation of gelatin, which can disrupt the regular lattice structure of CA, leading to a more amorphous phase. The transition to a more amorphous structure in the L/P 1 scaffold suggests that higher liquid content during scaffold preparation hinders the formation of well-ordered crystalline domains. Faster disintegration rates are characteristic of amorphous scaffolds, which may be favorable in situations where tissue regeneration is needed in conjunction with scaffold resorption. 25

Reducing crystallinity may enhance the material's resorbability, making it more suitable for tissue engineering applications where scaffold degradation is required to facilitate tissue regeneration. However, we acknowledge that reduced crystallinity may impact mechanical stability, a factor not assessed in this study. Future research will incorporate mechanical testing, such as compressive strength analysis, to evaluate the tradeoffs between enhanced bioactivity and structural stability.

### Structural and Morphological Insights from SEM Analysis

The SEM images visualize the scaffold's surface structure, revealing differences across the three L/P ratios. The L/P 0.5 scaffold exhibited the roughest surface with craters distributed more evenly. The L/P 0.8 and L/P 1 scaffolds demonstrated reduced roughness, with the L/P 1 scaffold showing a more compact and less crater structure. SEM images demonstrated the surface morphology of the scaffolds, with L/P 0.5 showing the roughest texture compared with the smoother surfaces of L/P 0.8 and L/P 1. These observations are consistent with prior studies that suggest the liquid content in the scaffold preparation plays a pivotal role in determining pore size and distribution. 26 A higher L/P ratio facilitates greater ice crystal formation during lyophilization, which, upon sublimation, leaves larger voids within the scaffold. Porosity is critical in scaffolds for tissue engineering, as it promotes nutrient exchange, cell migration, and vascularization. 27

A higher magnification should be studied further to identify homogen and well-defined pores needed for tissue engineering. SEM analysis of scaffolds is usually conducted at higher magnifications (e.g., 10,000 × ) to observe fine microstructural details. In this study, the 30× and 250× magnifications were selected to provide critical insights into the macrostructural properties of the scaffolds. At 30× and 250× magnification, the macrostructure of scaffold surface is clearly visible, offering valuable information regarding the influence of liquid content affecting structure of scaffolds. These parameters are essential for early-stage study of biomaterial for tissue engineering applications, where the overall architecture significantly influences biological outcomes. The choice of magnification also reflects the intended application of these scaffolds, focusing on structural integrity and mechanical stability, which are particularly relevant in load-bearing tissues or in cases requiring gradual scaffold resorption. 28 The limitation of this study highlights the potential need for increasing the BG percentage in future scaffold designs to enhance bioactivity and improve pore interconnectivity. Quantitative porosity analysis using image analysis software or complementary techniques such as porosimetry is also recommended for future studies to provide deeper insights into scaffold suitability for tissue engineering applications. 29

### Comparison with Original CA

The comparison between the original CA and CA-BG scaffolds provides valuable insights into how the addition of BG affects the material properties. In this study, the original CA, characterized by its chemical and crystalline structure, serves as a stable base material. Incorporation of CA with BG introduces flexibility and most likely will enhance the scaffold's biological function. The shifts in FTIR peaks and the reduction in crystallinity observed in XRD patterns highlight the interaction between CA and BG, suggesting that gelatin not only influences the mechanical properties but also affects the scaffold's chemical behavior. This transformation from a purely ceramic scaffold to a composite (mixture of CA and BG) material with both organic and inorganic components could offer significant advantages for tissue engineering, especially endodontic regeneration, with the complexity of dentin and pulp tissue. 30 31

### Role of Bovine Gelatin in Bioactivity

BG, derived from collagen, plays a critical role in enhancing the bioactivity of CA-BG scaffolds. Gelatin provides a bioactive surface with cell-adhesive motifs, promoting cell attachment, proliferation, and differentiation, which are vital for tissue regeneration. 19 Mechanistically, gelatin's carboxyl and amine groups interact with cellular receptors such as integrins, facilitating adhesion and intracellular signaling pathways that regulate cell behavior. 17 Furthermore, gelatin's ability to form hydrogels creates a three-dimensional microenvironment conducive to nutrient exchange and cellular migration, which are essential for effective tissue repair. These properties underscore gelatin's complementary role in enhancing the biological functionality of CA-based scaffolds.

### Comparison with Other Scaffolds

This section compares the novel CA-BG scaffold against other scaffolds traditionally used in endodontic tissue engineering, including collagen-based scaffolds, synthetic polymers, and bioceramics.

Collagen-based scaffolds have been extensively used in scaffolds for endodontic regeneration due to their biocompatibility and ability to replicate the natural extracellular matrix. Collagen scaffolds have successfully enhanced cellular attachment and facilitated pulp tissue regeneration. 32 However, collagen-based scaffolds typically suffer from limited mechanical strength and rapid degradation, leading to insufficient support for complete tissue regeneration. 33 In contrast, the CA-BG scaffold offers enhanced mechanical properties through the integration of CA, which provides greater structural integrity and a controlled resorption rate, thereby supporting long-term tissue healing. While collagen scaffolds primarily focus on bioactivity, the CA-BG scaffold combines bioactivity with osteoconductivity, delivering dual benefits for both soft and hard tissue regeneration in endodontic regeneration.

Synthetic polymer scaffolds like PGA, PLA, and poly (lactic-co-glycolic acid) are widely utilized in tissue engineering due to their adjustable degradation rates and ease of fabrication. However, their limited bioactivity raises concerns about their efficacy in complex tissue regeneration, particularly in dentin and pulp regeneration, where biological cues are critical. 34 The CA-BG scaffold, incorporating a natural gelatin component, offers superior bioactivity compared with synthetic polymers, promoting cell attachment and proliferation. Additionally, synthetic polymers often produce acidic byproducts during degradation, impairing cellular viability. In contrast, the CA-BG scaffold is naturally resorbable, avoiding the release of harmful byproducts and making it more suitable for the root canal environment, where pH balance is essential for tissue regeneration. 35

Hydrogels are often investigated as injectable scaffolds for endodontic regeneration because they can adapt to the irregular shapes of root canals and encapsulate cells, providing a supportive environment for tissue growth. 36 Hydrogels made from materials such as hyaluronic acid, alginate, and chitosan have demonstrated efficacy in promoting angiogenesis and pulp tissue formation. However, the main drawback of hydrogels is their lack of mechanical strength, which limits their effectiveness in regenerating hard tissues like dentin or bone. The CA-BG scaffold addresses this shortcoming by incorporating CA, which offers the mechanical support necessary for regenerating both soft and hard tissues. While hydrogels excel in supporting angiogenesis and pulp tissue regeneration, the CA-BG scaffold provides a more comprehensive solution by supporting both dentin and pulp regeneration. This strategy of employing natural and synthetic scaffolds has proven effective in promoting dental pulp regeneration. 37

Calcium phosphate scaffolds, including HA and β-TCP, are well-known for their osteoconductive properties, making them ideal for bone and dentin regeneration. These materials are frequently used in endodontic applications due to their compositional similarity to natural bone and dentin. 4 But in endodontic regeneration, which required more biological cues to promote soft tissue regeneration, calcium phosphate materials need incorporation with other biomaterials to promote cellular attachment and proliferation. The CA-BG scaffold offers an advantage over conventional calcium phosphate scaffolds by incorporating BG, which provides the bioactivity necessary for soft tissue repair. Collagen promotes the adhesion of dental pulp stem cells and facilitates angiogenesis, both of which are critical for successful pulp regeneration. 8 34 The bioactivity of BG and the osteoconductive qualities of CA should work together to promote both soft and hard tissue regeneration in the root canal environment. Bioactive glass scaffolds are valued for their ability to release biologically active ions, such as calcium and phosphate, which can stimulate and promote tissue regeneration. 38 Bioactive glass is also well-known for enhancing angiogenesis and hard tissue formations. 39

The CA-BG scaffold and bioactive glass have several basic components in common, notably its capacity to release calcium ions that will aid tissue regeneration. The CA-BG scaffold's potential to generate a tunable structure, providing both mechanical stability and controlled resorbability, renders it highly suitable for more complex tissue regeneration in the intricate and challenging environment of the root canal. By comparison, the CA-BG scaffold combines the bioactivity of gelatin with the structural integrity and osteoconductive properties of CA, offering controlled resorption that aligns with the tissue healing process. Although our study focuses on qualitative data, future work could further quantify parameters such as degradation rates and compressive strength to substantiate the advantages of CA-BG scaffolds.

### Implications for Endodontic Regeneration

Biodegradation is a critical factor influencing scaffolds' long-term stability and functionality in endodontic applications. 40 The gradual resorption of CA ensures that the scaffold degrades together with new tissue formation, avoiding premature loss of mechanical support. 41 42 However, rapid gelatin degradation could limit the scaffold's long-term bioactivity, necessitating optimization of the CA-BG ratio for sustained performance. Balancing biodegradation with structural stability and bioactivity is essential for successfully integrating CA-BG scaffolds into the dentinal walls and surrounding tissues. Future studies will evaluate these dynamics through *in vivo* testing to confirm the scaffold's suitability for endodontic regeneration.

The findings of this study suggest that CA-BG scaffolds have significant potential for endodontic regeneration. Further research is necessary to determine the porosity of the scaffolds to determine their capacity for cell infiltration and nutrient diffusion, both of which are essential for the effective regeneration of tissue in the dental pulp. 31 The moderate reduction in crystallinity with the addition of BG may promote scaffold resorption at a rate conducive to tissue regeneration, ensuring that the scaffold gradually degrades as the natural tissue regenerates. 43 Moreover, the bioactivity introduced by gelatin is an important factor in the scaffold's ability to support cell attachment and proliferation. 44 In the context of endodontic regeneration, where more complex of soft and hard tissue engineering is required, the CA-BG scaffold offers a promising balance of structural integrity, resorbability, and biological function.

Endodontic regeneration on immature teeth also raises doubts about the survival of apical papilla stem cells (SCAPs). SCAPs in the apical papilla serve as a reservoir of mesenchymal stem cells critical for root development and dentin–pulp complex formation. However, the survival of SCAPs during tooth infection remains challenging in endodontic regeneration. 45 46 The interaction of CA-BG scaffolds with stem cells, particularly stem cells from the apical papilla (SCAPs), can play a pivotal role in their regenerative potential. The CA-BG scaffold offers a bioactive and osteoconductive environment that could support SCAP adhesion and differentiation through its controlled ion release and bioactive properties. By promoting epithelial–mesenchymal interactions, the scaffold could enhance SCAP-mediated tissue regeneration. Future studies must evaluate the scaffold's ability to maintain SCAPs viability and functionality in infected environments, which is essential for successful endodontic regeneration.

## Strengths and Limitations

This study provides foundational insights into the structural and bioactive properties of CA-BG scaffolds, highlighting their potential in tissue engineering and endodontic regeneration. Characterization using FTIR, XRD, and SEM gives insights into the scaffold's chemical and structural attributes. However, the study is exploratory and limited by the qualitative approach, the absence of *in vivo* testing, and the lack of mechanical strength assessments. These limitations suggest the need for future research to evaluate scaffold performance quantitatively under physiological conditions.

## Future Perspectives

Future research should optimize the CA-to-BG ratio to balance bioactivity, mechanical stability, and biodegradation rates. *In vivo* studies are needed to confirm the scaffold's efficacy in endodontic regeneration. Additionally, incorporating advanced analytical techniques such as porosimetry and higher-resolution SEM imaging could provide deeper insights into pore interconnectivity and its role in cell infiltration. These studies will be critical for translating CA-BG scaffolds into clinical applications, specifically endodontic regeneration.

## Conclusion

This study demonstrates the potential of CA-BG scaffolds as a promising material for endodontic tissue engineering. The L/P 1 ratio was identified as the most favorable due to its structural stability and retained crystallinity. These properties suggest that the scaffold could be particularly effective in promoting dentin regeneration, where structural support and controlled resorption are critical. Additionally, the bioactivity introduced by gelatin could enhance pulp regeneration by facilitating cellular attachment, proliferation, and differentiation. The CA-BG scaffold holds potential for applications in different stages of endodontic tissue repair. It could serve as a bioactive platform for pulp tissue regeneration in its early stages, promoting vascularization and cellular migration. In later stages, the scaffold's osteoconductive properties may enhance dentin tissue formation by integrating with dentinal walls and restoring structural integrity. However, additional quantitative studies with larger sample sizes and *in vivo* testing are needed to confirm its effectiveness and optimize its composition for long-term clinical use.
